# Evaluation of a Telehealth Paediatric Asthma and Allergy Clinic Patient Follow-Up During the COVID-19 Pandemic

**DOI:** 10.3390/children11121507

**Published:** 2024-12-11

**Authors:** Sarah J. Prior, Gaylene Bassett, Emmeline Van Beek, Claire Griffiths, Smriti Sinha, Shrima Shrestha, Sukhwinder Singh Sohal, Heinrich Christoph Weber

**Affiliations:** 1 Tasmanian School of Medicine, University of Tasmania, Burnie, TAS 7320, Australia; 2 Tasmanian Health Service—Northwest, Burnie, TAS 7320, Australia; gaylene.bassett@ths.tas.gov.au (G.B.); claire.griffiths@ths.tas.gov.au (C.G.); smriti.sinha@utas.edu.au (S.S.); shrima.shrestha@ths.tas.gov.au (S.S.); heinrich.weber@utas.edu.au (H.C.W.); 3 Rural Clinical School, University of Tasmania, Burnie, TAS 7320, Australia; vanem@utas.edu.au; 4 Respiratory Translational Research Group, Department of Laboratory Medicine, School of Health Sciences, College of Health and Medicine, University of Tasmania, Launceston, TAS 7248, Australia; sukhwinder.sohal@utas.edu.au

**Keywords:** pandemic, outpatient clinic, rural, respiratory disease, patient experience

## Abstract

Background/Objectives: The COVID-19 pandemic created many challenges for health services, particularly during lockdown periods. Asthma is one of the most common childhood conditions, and children with asthma are generally cared for in an outpatient setting. In regional Northwest Tasmania, during the COVID-19 lockdowns, asthma and allergy outpatient clinic services were transferred to a virtual platform in the form of telehealth appointments for routine consultations. This study aims to explore the patient and family perspectives of a telehealth outpatient asthma review service in a regional area. Methods: We utilised a mixed-methods approach, interviewing parents and children who attended the clinic via telehealth and collating routine clinical audit data collected during the same period. Results: Sixty-three patient reviews were audited, with a mean age of 9.79 years. Since their last review, 13% visited their GP, 9% presented to the ED, 6% were hospitalised, and 19% had changes in their treatment plan. Interviews highlighted three main themes: (i) telehealth is a convenient strategy for routine asthma and allergy review appointments, (ii) barriers associated with using telehealth are a minor concern for parents of children with asthma and allergy routine appointments, and (iii) shared decision-making between clinicians and parents of children with asthma and allergy around the type of appointment required is valued. Conclusions: We demonstrated that the benefits of telehealth include implementing treatment changes and facilitating access to care for children living in rural and remote areas.

## 1. Introduction

The COVID-19 pandemic resulted in almost half a billion cases and in an excess of 6 million deaths worldwide [1]. This global pandemic led to the closing of borders and lockdowns that significantly impacted healthcare delivery and the future planning of health services. During the pandemic, the healthcare emphasis was on acute care management with routine health service delivery not considered a priority. This latter situation particularly affected chronic care management as patients and their families avoided attending scheduled routine outpatient clinic appointments.

Asthma is the most common chronic condition in childhood and the most common reason for childhood hospital admissions [2]. The prevalence of asthma in Australia is 11%, which equates to about 2.7 million people living with asthma in Australia [3]. This is high compared to world standards [4]. Asthma adversely affects a child’s quality of life and is associated with significant comorbidities such as obesity, gastro-oesophageal reflux, and mental health issues [5]. Paediatric patients with asthma need regular monitoring to maintain good asthma control, prevent asthma attacks, and manage any comorbidities. Asthma is still significantly undertreated as only 38% of individuals with asthma receive appropriate care, in line with current evidence-based guidelines [6]. Routine outpatient clinics were disrupted by the pandemic, and as a result, patients were not always able to receive their regular care. Alternate means of follow-up, such as telehealth, were considered an ideal alternative to provide ongoing routine care.

Telehealth is defined as the delivery of health services via a range of information technology tools such as telephone, video conferencing, or internet platforms. The aim of telehealth is to improve patient care by removing barriers such as access [7,8] and reducing costs to patients and healthcare organisations [9]. The emergence of COVID-19 has had a significant impact on many healthcare services with many suspending face-to-face consultations in favour of telehealth to mitigate the risk of the spread of SARS-CoV-2 virus and its consequences. However, telehealth for paediatric patients can present some unique challenges. One of the challenges for all paediatric consultations is communicating with patients of varying ages and levels of understanding. The use of telehealth could further hinder this communication between clinicians and paediatric patients and introduce the possibility for technical problems during consultations. Studies prior to COVID-19 suggested that telehealth (prior to COVID-19) results in similar health outcomes for chronic patients monitored via telehealth when compared with in-person reviews [10] and general patient satisfaction [11]. Brownlee et al. [12] suggested that teleconsultations are a reliable and robust method of clinical review for paediatric surgical conditions and Smith et al. [13] found that diagnosis and management plans for paediatric ear, nose, and throat (ENT) surgery made by video conferencing were in agreeance with those made through face-to-face consultations. These studies utilised both parent(s) and children during consultations and identified several enablers and barriers including cost-saving and the importance of physical examination in some cases.

Telehealth has been utilised previously as a tool for asthma review, and Romano et al. [14] found that children receiving telehealth visits with an asthma care specialist increased their number of symptom-free days by 83%, and their mean asthma symptom scores reduced by 44%. Telehealth access to an asthma specialist also results in better symptom control, improved functional status, and a reduction in the number of asthma attacks [15,16]. Although the clinical efficacy of telehealth has been demonstrated before, a number of questions remain with regards to the patient’s perspective of this means of consultation.

The aim of this project is to explore the patient and family perspectives of a telehealth outpatient asthma review service in a regional area.

## 2. Materials and Methods

### 2.1. Setting and Participants

Northwest Tasmania is a regional area with a dispersed population of approximately 120,000 people and is serviced by two public hospitals, each with a paediatric outpatient asthma clinic. In regional Tasmania, the prevalence of childhood asthma is higher than the national average [3]. The emphasis in managing asthma is to achieve control to improve the quality of life of patients and reduce the exacerbation rate. Clinical staff working within the paediatric outpatient setting in regional Tasmania have determined that over 650 patients are on a public waiting list requiring routine asthma review. Therefore, a decision was made to contact these patients (their parent(s) and or/guardians) via telephone to conduct routine asthma/allergy review questionnaires (Asthma Review Proforma, Supplementary Materials) as per standard outpatient practice.

Participants for this study included all children (and/or their parents and guardians) with asthma or allergies who regularly attend the one of the two paediatrics asthma outpatient services in Northwest Tasmania and asthma patients requiring recent hospitalisation. These patients had recently been contacted by the clinical team to complete an asthma health questionnaire over the phone (telephone consultation) as part of their routine care during the COVID-19 pandemic.

This project was approved by the Tasmanian Human Research Ethics Committee, reference H0021642. Consent was obtained during the telehealth consultation from the parent(s)/guardian(s) to be contacted by a researcher for a follow-up interview evaluating the service they received. Upon gaining consent, their identification and contact details were collected. Consent was reconfirmed prior to starting data collection.

### 2.2. Procedure

#### 2.2.1. Clinical Audit Data

Clinical audit data were collated from questionnaires that were undertaken as part of the routine follow-up phone calls of asthma/allergy patients (See Asthma Review Proforma—Supplementary Materials). The questionnaire is an internal health service tool that is routinely used to collect information for patient review. It includes six main sections: demographic data, clinical data, triggers, treatment, comorbidities, and plan. The objective of the clinical audit data was to determine the proportion of patients requiring a change in their asthma treatment plan.

#### 2.2.2. Interviews

Consenting participants were contacted by one of two members of the research team via telephone. Each participant was provided with further standard information about the project and asked for verbal consent if they were still interested in discussing their telehealth consultation experience for evaluation purposes. All interviews were audio-recorded. Parent(s)/guardian provided most of the information. However, if the child (patient) wished to contribute, the researcher spoke with the child in the presence of their parent/guardian about their experience.

Each interview was semi-structured with a focus on three main areas: concerns around being able to see a healthcare professional during the COVID-19 pandemic, perceptions of the telehealth service, and preferences for future appointments with healthcare professionals.

### 2.3. Data Analysis

#### 2.3.1. Clinical Audit Data

All clinical data from the questionnaires were collated and analysed in Microsoft Excel as descriptive data.

#### 2.3.2. Interviews

All audio data were transcribed within the team and analysed through the qualitative method of thematic analysis. Analysis was performed in two parts based on an analytic tool [17]. The first part involved the development of thematic networks and involved three main steps:

1. Breaking down the text: developing the thematic network;
2. Exploring the text: describing the thematic network;
3. Integration: interpreting the patterns within the thematic network.

Three members of the research team were involved in the development of the thematic networks by independently coding the transcribed data and identifying basic themes. The researchers reviewed and compared the basic themes and used this information to ascertain and agree on final thematic networks.

The second part of the analysis included identifying organising themes from patterns observed as part of the basic themes and utilising these themes to identify global, overarching themes within the interview data. Following a check, re-check review, and further analysis, the research team identified the final, global, overarching themes.

## 3. Results

The results suggest that there are several factors that influence the satisfaction and experience of patients and/or their guardians, in response to telephone consultations with their asthma and/or allergy healthcare provider.

### 3.1. Clinical Audit Data

Sixty-three patient questionnaires from planned review appointments were audited. The average age of the children was 9.79 years with a slightly higher number of male participants (60%). Just over half of the participants audited were contacted during the COVID-19 specified period, with a small number unable to be contacted (8%). Thirty-one participants (49%) required a face-to-face review. Table 1 presents the participant demographics including co-morbidities.

Of those participants who were able to be contacted and had their review via telehealth, over half (53%) were awaiting allergy testing and would need a follow-up face-to-face appointment. Twenty-two percent had worsening symptoms in the three months prior to their review although only thirteen percent visited their GP. Nine percent presented to the ED, and of these, sixty-six percent were hospitalised. Nineteen percent of the participants reported a change in treatment since their last review as advised by their GP, ED physician, or paediatrician. Table 2 shows the clinical data that were collected during the telehealth planned review consultations.

### 3.2. Interviews

Thirteen interviews were conducted by two members of the research team who spoke to the maternal parent of each child. Three children also participated in interviews. Five (39%) children were being reviewed as asthma patients, three (23%) for allergies, four (31%) for both asthma and allergies, and one child (7%) had not been diagnosed with either asthma or allergies but was being reviewed for potentially having both. The age range of the children was 1 year to 15 years old (M = 6.4 years).

There were three global, overarching themes that were derived from the interview data. These are explained in further detail below.

#### 3.2.1. Theme 1: Telehealth Is a Convenient Strategy for Routine Asthma and Allergy Review Appointments

Telehealth consultations were reported as a convenient way for more than half of the participants to meet with their healthcare professionals. Some of the limiting factors for telehealth consultations that were identified included mobility limitations, large families, rural families, and working families. Saving time was also a key factor, particularly around not having to wait in a waiting room and travel time.

*“When [child] is well and does not require physical tests, phone appointment provide similar quality information in more convenient and easier manner”*

P2

Although it was noted that there were still some delays around appointment times, having to wait for the healthcare professionals to become available, this was seen as a minor issue and did not significantly affect the participants as they were able to continue with other things whilst waiting.

#### 3.2.2. Theme 2: Barriers Associated with Using Telehealth Are a Minor Concern for Parents of Children with Asthma and Allergy Routine Appointments

Although some barriers around telehealth consultations were identified, the participants suggested that these were only a minor concern, as the convenience of the appointment outweighed these barriers. Children did not always attend telehealth appointments, but they did attend face-to-face. When children did attend the telehealth appointments, the participants reported that it was harder to get their children to engage with the healthcare professional(s). The participants felt that this lack of engagement did not affect the outcomes of their appointments. Similarly, the participants suggest that telehealth consultations were led by the healthcare professional, whereas parents are more likely to lead the conversation in a face-to-face appointment.

*“The doctor talks more over the phone”*

P6

Technical barriers were also identified, such as the technology not working for either the patient or the healthcare professional, having multiple calls from multiple healthcare professionals for the same appointment, and the participants not feeling comfortable answering a private number.

A further barrier identified was the ability to retain information that was provided over the phone. Three participants highlighted that they found it more difficult to remember what had been discussed and the outcomes from their consultations when they did not see their healthcare professional face-to-face. The participants indicated that they felt most information was covered, and the quality was the same, when compared to a face-to-face consultation, but they had more difficulty remembering it following a telehealth call.

*“Information is probably the same but harder to retain over the phone. I would be listening more over the phone and not talking which would make it harder to ask question”*

P11

#### 3.2.3. Theme 3: Shared Decision-Making Between the Clinician and Parents of Children with Asthma and Allergy Around the Type of Appointment Required Is Valued

Whilst the participants highlighted a number of benefits of telehealth appointments, half of the participants also suggested that they would like to be provided with an option for face-to-face as well. In particular, if they felt that their child(ren) needs physical tests or a physical examination, or if there were other concerns, they would like to be seen in person in the first instance rather than be assessed via telehealth and then have to schedule a face-to-face appointment.

*“I would like to have a phone appointment as an option but would also like face-to-face appointments as well”*

P3

There was also some concern about delayed or missed review appointments if telehealth was not an option during times where patients were unable to visit the healthcare practice.

*“I was concerned that he would miss his asthma review, I just took action myself and upped his preventer and kept on top of things before I had another appointment.”*

P1

Similarly, it was reported that, if there was a pre-existing relationship between the child and the healthcare professional, telehealth appointments would be favourable as engagement would not be as much of an issue.

*“The tele appointment wasn’t with his normal paed either so if it as with the normal paed it would have been different”*

P7

Generally, the participants felt valued during their telehealth consultations, although a small number of the participants highlighted that their children were not present at their telehealth appointments as they did not know this was an expectation.

*“I felt very valued as a parent.”*

P8

## 4. Discussion

This study has demonstrated the usefulness of telehealth consultations for paediatric asthma and allergy clinic patients waiting for routine review appointments in a regional area. In a substantial proportion of cases, telehealth provided an opportunity to adjust treatment accordingly, which is consistent with previous research that suggests telehealth as a suitable alternative to in-person consultations [12,13]. This study further outlines the experiential aspects of telehealth requiring consideration when managing paediatric chronic disease, such as childhood asthma. The value, barriers, and facilitators of telehealth services for childhood asthma have been reported recently as part of an integrative review [18] with a focus on the need for more qualitative research. Whilst Chike-Harris et al. [18] suggest that health care disparities may increase with the use of telehealth in childhood asthma, our findings suggest that it provides improved access and equity for people living in regional areas.

In our study, a substantial proportion of patients required a change in treatment, more specifically, an escalation of treatment which highlighted the efficacy of telehealth in this space. Without this option, it is likely that escalation in treatment for our participants would have been delayed with potential serious consequences. Similar to our study, the efficacy of telehealth in managing patients with asthma has been well demonstrated [12,13], suggesting that the clinical benefit of telehealth consultations could likely be extended to the management of children with other chronic medical conditions. This study demonstrates that telehealth services facilitate access to care for children living in rural and remote areas, such as our setting, and was particularly useful during the COVID-19 lockdown when access to many healthcare services was limited.

This study further provides insight into the patient’s experiences of telehealth. The Australian government has continued to provide some financial support via Medicare for ongoing telehealth services in public hospitals post pandemic. Therefore, it is important to understand the patient and parent perspectives around the use of telehealth for routine outpatient asthma review. A case study by Galway et al. [19] describes the journey of an asthma review patient via telehealth services, focusing on education around the use of an inhaler and a management plan moving forward. In this case, as with many other telehealth consultations, direct engagement with the child was limited. The consultant relied mostly on information provided by the parent. This case also highlighted that telehealth works best when there is a pre-existing relationship between the patient, parent, and treating clinicians, which is consistent with our findings [19]. This type of relationship then allows more effective communication, a better flow of information, and better understanding of non-verbal cues in video conferencing. In some cases of our study, the patient (child) was not present during the telehealth consultation, meaning the healthcare professional was solely relying on information from the parent(s). Acknowledgement that some information may be missing from these types of consultations is a limitation of telehealth, particularly in this population.

Brownlee et al. [12] highlighted the need for face-to-face consultations when a physical examination is required. Our findings further reiterate the importance of face-to-face appointments when physical examinations and tests are scheduled, with parents raising concerns about missing or delaying physicals tests such as allergy testing and lung function tests. As mentioned above, most parents would like to be given the option of telehealth or face-to-face appointments to ensure that, when their child needs to be examined or tested, they can attend in person. A hybrid model of care for paediatric patients with a combination of telehealth and face-to-face appointments was proposed as a potential solution to social distancing during COVID-19 [20] but is also relevant for regional areas where geography can limit access.

This study took place at the in the Northwest region of Tasmania, an area that is classified as outer regional by the Australian Bureau of statistics and also provides health care services for remote areas of Tasmania. Rurality is one of the biggest factors that reduces access to healthcare. People who live in rural areas have higher travel distances and longer travel times to their nearest hospital and GP practice when compared to those who live in metropolitan areas [21]. With these greater travel distances come several consequences for people living in rural areas, including a higher financial burden to traveling to health care appointments and reduced or no public transport options. Many parents also expressed that telehealth appointments were more convenient particularly when their child had good symptom control and up-to-date testing. Parents expressed that telehealth was less time consuming, easier to attend, and led to less disruptions to their child’s schooling and their work. Apart from the convenience of telehealth, it also facilitated access to services, especially for those living in regional and rural areas, as in our circumstances. This physical barrier to access can often lead to poorer outcomes for patients and poorer attendance to routine follow-up appointments [22].

A limitation is that we could not determine if the change in treatment ultimately resulted in clinical benefit. Another limitation is the lack of use of objective measures, such as lung function tests, to assess asthma control. Symptom-based questionnaires are considered the standard way of evaluating asthma control in daily practice [23]. The correlation between symptom scores and lung function is variable. A more recent prospective study suggests that lung function tests could identify more children at risk of asthma exacerbations [24]. While this does not undermine this study’s results, additional lung function tests might have identified more patients with poor asthma control. A further limitation of this study is that we largely relied on the opinion of the parent to express their opinion of the telehealth interview process, which is a limitation experienced in other similar paediatric studies. A future consideration would be to obtain the opinion of the child, especially those of school age.

## 5. Conclusions

We demonstrated that the benefits of telehealth include implementing treatment changes and facilitating access to care for children living in rural and remote areas. This was particularly pertinent during the COVID-19 lockdown. A qualitative analysis further confirmed the adequacy of telehealth services as an appropriate and acceptable means of healthcare delivery and provides suggestions for improved care.

## Figures and Tables

**Table 1:** Demographic Information (1 April 2020–31 May 2020).

Variable	Male N = 38	Female N = 25	Total N = 63
Average age	10.33 years	8.94 years	9.79 years
Number of contacts made	15 (39%)	17 (68%)	32 (51%)
Could not be contacted	3 (8%)	2 (8%)	5 (8%)
Number of patients who required face-to-face review			31 (49%)
**Comorbidities**			
Eczema	8 (21%)	4 (16%)	12 (19%)
Allergic rhinitis	13 (34%)	6 (24%)	19 (30%)
Anaphylaxis	6 (16%)	2 (8%)	8 (13%)
Food allergy	7 (18%)	4 (16%)	11 (17%)
Snore or have disturbed sleep	8 (21%)	3 (12%)	11 (17%)

**Table 2:** Clinical data collected during telehealth consultations.

Clinical Data	Male N = 15	Female N = 17	Total N = 32
ED presentation since clinic visit in the last 3 months	1 (6%)	2 (12%)	3 (9%)
Oral corticosteroid usage in the last 3 months	2 (13%)	3 (18%)	5 (16%)
Oral antibiotic usage in the last 3 months	3 (20%)	1 (6%)	4 (13%)
Hospitalised in the last 3 months?	2 (13%)	0	2 (6%)
Are symptoms worse in the last month?	5 (33%)	2 (12%)	7 (22%)
Did you visit your GP in the last 3 months for asthma treatment?	3 (20%)	1 (6%)	4 (13%)
**Plan**			
Changes in Treatment	4 (27%)	2 (12%)	6 (19%)
Planned review face-to-face (in person)	12 (80%)	12 (71%)	24 (75%)
Telehealth planned review	1 (6%)	2 (13%)	3 (9%)

## Data Availability

The original contributions presented in the study are included in the article, further inquiries can be directed to the corresponding author.

## Supplementary Materials

The following supporting information can be downloaded at: https://www.mdpi.com/article/10.3390/children11121507/s1, Asthma Review Proforma.
