# Supplementary material for: Evaluation of a Telehealth Paediatric Asthma and Allergy Clinic Patient Follow-Up During the COVID-19 Pandemic
Source: Children (Basel). 2024 Dec 11;11(12):1507. doi: 10.3390/children11121507 (PMC11674270; doi:10.3390/children11121507)
Supplement: Supplementary file 1 [file children-11-01507-s001.zip › children-3342326-supplementary.pdf]

Supplementary Materials
Paediatric Asthma Outpatient Review Proforma

**DOB**	**PATIENT NAME**	**UR NUMBER:**
Click or tap to enter a date.		

Today's date: Click or tap to enter a date.	Last Clinic visit date: Click or tap to enter a date.
Adult details (Name):	
Parent:	Guardian:

**CLINICAL**			
ED presentation since clinic visit in last 3 months?	Yes ☐	No ☐	If yes, number
Oral corticosteroids usage in last 3 months?	Yes ☐	No ☐	If yes, number
Oral antibiotics usage in last 3 months?	Yes ☐	No ☐	If yes, number
Hospitalised in last 3 months?	Yes ☐	No ☐	If yes, number
Are symptoms worse in the last month?	Yes ☐	No ☐	If yes, number
Did you visit your GP in last 3 months for asthma treatment?	Yes ☐	No ☐	If yes, number

**TRIGGERS**	
What do you think are the major TRIGGERS for your or your child's asthma?	☐ URTI<br>☐ House dustmite<br>☐ Grass<br>☐ Animals<br>☐ Smoke<br>☐ Stress<br>☐ Other (please state):

**TREATMENT**	
Inhaled corticosteroid:	Yes ☐ No ☐ If yes:<br>☐ Fluticasone propionate (Flixotide)__________(*dosage*)<br>☐ Budesonide (Pulmicort)<br>☐ Ciclesonide<br>☐ Fluticasone propionate/salmeterol (Seretide)<br>☐ Budesonide/eFormoterol<br>☐ Other (please state):
Montelukast (Singulair)	Yes ☐ No ☐

Are you using your preventer medications more regularly in the last month?	More ☐ No change ☐ Less ☐
How many **days** a week did your child use their preventer?	Not at all ☐ <3 ☐ 3-5 ☐ 7 days a week ☐
Bronchodilator or salbutamol usage?<br>How many times a week did you use the reliever medication?	None ☐ <2 ☐ >2 ☐ everyday ☐
Do you have enough medications at home?	Yes ☐ No ☐
**COMORBIDITIES**	
Eczema Yes ☐ No ☐	Rx: Topical corticosteroid: Yes ☐ No ☐
Allergic rhinitis Yes ☐ No ☐	Rx: Intranasal corticosteroid: Yes ☐ No ☐
Anaphylaxis: Yes ☐ No ☐	Allergen?
Epipen prescribed? Yes ☐ No ☐	Epipen expiry date? Click or tap to enter a date.
Anaphylaxis action plan in date? Yes ☐ No ☐	
Antihistamine prescribed?	Yes ☐ No ☐
Alert disk?	Yes ☐ No ☐
Food Allergy?	Yes ☐ No ☐ If yes:<br>☐ Cow's milk ☐ Egg<br>☐ Peanut ☐ Tree nut<br>☐ Soya ☐ Other:_______________
Do you snore or have disturbed sleep	Yes ☐ No ☐
Any other medical condition?	Yes ☐ No ☐ If yes:<br>☐ ADHD ☐ ASD<br>☐ Other (please state):_________________________
Are you awaiting allergy tests?	Yes ☐ No ☐
Asthma control test (ACT)	https://www.asthmacontroltest.com/en-au/welcome/
**PLAN:**	
Changes in treatment:	Yes ☐ No ☐ If yes:<br>☐ Stepping up ☐ Stepping down<br>Add treatment:<br>Treatment change:

Asthma written action plan:<br>Do you have an up to date WAAP?	Yes ☐ No ☐
Plan for follow up:	
Would you mind if we call you in the next month or two to ask more questions related to your son/daughter or yourself?	Yes ☐ No ☒
Any comments:	
**CLINICIAN NAME, TITLE, SIGNATURE AND DATE**	
Name:	Title:
Signature:	Date:
